# From Village to Clinic: Structural Barriers and Intersecting Challenges in Maternal Healthcare Access in Rural Nepal

**DOI:** 10.3390/ijerph23040454

**Published:** 2026-04-01

**Authors:** Lalita Kumari Sah, Eleni Hatzidimitriadou, Prabhu Sah

**Affiliations:** 1 Faculty of Health and Social Care, University of Bradford, Bradford BD7 1DP, UK; 2 Faculty of Medicine, Health and Social Care, Canterbury Christ Church University, Canterbury CT1 1QU, UK; eleni.hatzidimitriadou1@canterbury.ac.uk; 3 District Hospital Ilam, Ilam Municipality, Koshi Province, Ilam 57300, Nepal; drprabhusah@gmail.com

**Keywords:** maternal health services, social determinants of health, intersectionality, perinatal health equity, structural barriers

## Abstract

**Highlights:**

**Public health relevance—How does this work relate to a public health issue?**

- Examines how geographic isolation, inadequate infrastructure, and poverty intersect to shape maternal health experiences in rural Nepal.
- Applies the Social Determinants of Health framework and intersectionality to understand systemic barriers to maternal care.

**Public health significance—Why is this work of significance to public health?**

- Reveals persistent gaps in policy implementation, including transport, accommodation, and financial protection for pregnant women.
- Highlights the compounded disadvantage faced by women from rural and marginalised communities, impacting equity in maternal health outcomes.

**Public health implications—What are the key implications or messages for practitioners, policymakers and/or researchers in public health?**

- Advocates for decentralisation of maternal health services and improved rural transport infrastructure.
- Recommends implementation of maternity waiting homes and inclusive financial protection schemes to reduce systemic inequities.

**Abstract:**

This study explores the lived experiences of pregnant women in rural Nepal navigating maternal healthcare amidst intersecting structural barriers. Using the Social Determinants of Health framework and intersectionality, we examine how geographic isolation, inadequate infrastructure, and economic hardship compound risks to timely and safe maternal care. Twenty in-depth interviews were conducted at a district hospital in the eastern region of Koshi Province, Nepal. Four major themes were identified through inductive thematic analysis. These are: geographic vulnerability and transport challenges; gaps in rural maternal health provision; accommodation and institutional support deficits; and economic vulnerability and hidden costs of care. Findings reveal that poor road conditions, unreliable transport, and limited diagnostic services force women to undertake long, costly journeys, often requiring temporary relocation without institutional accommodation support. Despite policies such as the Safe Motherhood Programme, implementation gaps persist, leaving women to bear significant financial and emotional burdens. These experiences underscore systemic inequities in resource distribution and highlight the compounded disadvantage faced by women from rural and marginalised communities. To ensure equitable maternal healthcare, this study advocates for the decentralisation of health services and the implementation of inclusive financial protection policies tailored to the needs of women from rural and marginalised communities. To promote equitable maternal healthcare, we recommend strengthening rural health infrastructure, implementing maternity waiting homes, and expanding financial protection schemes tailored to vulnerable populations. This research offers critical insights for policymakers to address maternal health inequalities and advance Nepal’s progress toward Universal Health Coverage and Sustainable Development Goal 3 (Ensure healthy lives and promote well-being for all at all ages).

## 1. Introduction

Maternal health remains a critical public health concern in Nepal, particularly in rural and remote regions where access to timely and quality healthcare is often limited to women from disadvantaged backgrounds. In recent times, an increasing number of Skilled Birth Attendants (SBAs) have provided critical interventions that improved maternal and neonatal health and reduced maternal and neonatal mortality in Nepal [1]. However, distance from health facilities to home and inadequate transportation are known as significant barriers to the utilisation of SBAs, particularly in rural hilly regions of Nepal [2]. Evidence suggests that providing women with transportation incentives before they go to a facility for delivery and managing transportation options will increase service utilisation [3]. Recently, the Safe Motherhood Policy and the Skilled Birth Attendant Policy have notably improved maternal and child mortality rates in Nepal; however, there are ongoing arguments about the effective implementation of these policies, focusing on local needs, such as birth preparedness, financial incentives, free delivery services, and community post-partum care programmes and the targeting of women from lower socioeconomic backgrounds and rural regions [4]. The voices of rural women, as captured in this study, offer a compelling lens through which we explore the persistent structural and systemic barriers that shape maternal health experiences in Nepal. This study aims to explore how geography, infrastructure, health service availability, and socioeconomic factors intersect to shape rural pregnant women’s experiences of accessing maternal healthcare in Nepal.

### 1.1. Challenges in Health Service Access in Nepal

Nepal’s healthcare system follows a three-tier structure: primary care through health posts and primary healthcare centres (PHCCs), secondary care via district hospitals, and tertiary care in regional and central hospitals. While most comprehensive maternal services are concentrated at the secondary and tertiary levels, geographical isolation and unreliable transport systems remain major deterrents for rural women, preventing timely access to essential care [5]. Although the government has introduced programmes such as the Safe Motherhood Programme (also known as Aama Surakshya Programme) to decentralise services and expand maternal health coverage, implementation gaps remain a major challenge in rural areas. Evidence shows that, despite maternity services being officially free under this program, pregnant women and their families still incur substantial indirect costs, including expenses for food, transport, clothing, and medicines, alongside income loss during hospital stays [6]. Nepal’s unreliable public transport system makes travel during late pregnancy physically uncomfortable, and when combined with financial hardship and the limited scope of services at rural health posts, these barriers force expectant mothers to undertake long and difficult journeys just for basic care [7]. The World Health Organisation document on Delivering Quality Health Services: A Global Imperative for Universal Health Coverage emphasises that we can achieve Universal Health Coverage (UHC) by 2030 if we manage to provide integrated services within systems, not only infrastructure, medical supplies, and skilled healthcare providers in isolation, and instead both physical infrastructure and a professional, motivated health workforce that prioritises quality as we understand it to be effective, safe, timely, equitable, and efficient [8]. Primary care, such as health posts in remote villages, often lacks essential diagnostic tools, skilled personnel, and emergency obstetric care, forcing women to travel long distances to urban hospitals just for basic services [7]. In addition, evidence suggests that primary healthcare in the mountain and hill regions of Nepal is characterised by low standards and inadequate functioning, including misbehaviour by health professionals, local health governance failures, and the lack of cultural acceptance and integration of formalised maternal care [9]. As a result, women must make repeated trips to urban hospitals at both financial and emotional costs. These gaps in service availability reflect broader systemic inequities in the distribution of healthcare resources.

In Nepal, where financial hardship, quality of care, geographic and infrastructural barriers often prevent timely access to skilled obstetric care, many pregnant women from remote areas are forced to relocate to urban centres in the final weeks of pregnancy, which further exacerbates their financial and emotional burdens due to the lack of institutional support for temporary accommodation [10]. This often means staying with relatives or paying for lodging and food, which counts as a hidden cost of accessing maternity services and also adds financial strain and emotional stress. The absence of institutional support for temporary housing or accommodation for women from remote and disadvantaged communities who cannot afford accommodation in the town or near larger health service centres highlights a critical oversight in maternal health planning under the policy. The concept of maternity waiting homes (MWHs) is not new and was documented and reviewed nearly three decades ago by the World Health Organisation [11]. According to the document, MWHs are residential facilities near hospitals where high-risk or geographically isolated women can stay before delivery and also offer a practical, low-cost solution to bridge the gap in health service access. They are noted as most effective when integrated into a broader continuum of care, supported by skilled health professionals, and providing reliable emergency services. For Nepal, incorporating MWHs into the national maternal health policy and effective implementation could significantly improve maternal and neonatal outcomes, reduce preventable deaths, and promote equitable access to institutional delivery, especially in underserved regions and disadvantaged groups of women. For the first time, Nepal has recognised MWHs in its policy and strategic roadmap called ‘Safe Motherhood and Newborn Health Road Map 2030’ [12]. It aligns with Sustainable Development Goal (SDG) 3 and Nepal’s Safe Motherhood and Reproductive Health initiatives, highlighting the importance of maternity waiting homes (MWHs) as a strategic intervention to improve maternal and newborn health outcomes, particularly in remote and mountainous regions. However, the implementation responsibility is assigned to the local government, which could pose a challenge due to inadequate awareness and prioritisation of maternal health issues by the local government. There is already evidence of the success of MWHs in many developing countries, confirming that the intervention of MWHs reduces maternal mortality and stillbirth rates, and is very valuable for resource-constrained settings of developing countries [13,14]. However, it is important to explore through research why and under what conditions pregnant women in Nepal would seek such accommodation, as understanding these factors is central to designing effective policies and interventions that address the women’s needs. Given that many women in this study described difficulties securing temporary accommodation, the discussion of MWHs is included to contextualise these barriers within Nepal’s policy landscape.

### 1.2. Theoretical Framework

In this study, through the narratives of pregnant women, we aim to explore their experience of structural factors such as how geography, infrastructure, health service availability and poverty intersect to create compounded challenges for pregnant women in rural Nepal, including transport-related barriers and accommodation issues in the town, limited health services near the nearby health post, and financial concerns associated with the service access. To understand these complex and intersecting factors influencing maternal health service utilisation in rural Nepal, this study draws on the WHO Commission on Social Determinants of Health (CSDH, 2010) Conceptual Framework, which provides a structured model for examining how structural conditions shape health inequities, including access to healthcare, transportation, education, income, and housing which are the primary focus of this study [15]. In this study, the SDH framework helped guide our analysis by focusing attention on key determinants relevant to women’s experiences, such as the physical environment (road conditions and geography), access to health services (availability of diagnostic and emergency care), economic conditions (costs of travel, tests, and income loss), and social circumstances (housing, family support, and temporary relocation). These components directly supported our interpretation of how broader structural factors shaped women’s ability to access timely maternal healthcare in rural Nepal. While the SDH framework helped identify these structural determinants, the concept of intersectionality allowed us to explore how they interacted, particularly how geography, poverty, gender norms, and social support combined to create compounded challenges for rural women. Therefore, alongside SDH, we aim to apply the concept of intersectionality coined by Crenshaw [16,17] to explore how structural and systemic barriers intersect to affect women’s ability from rural areas to access safe and timely maternal care. As we acknowledge, there is a lack of studies that apply the intersectionality concept and examine how intersecting structural barriers, such as geography, infrastructure, and poverty, shape maternal health experiences in rural Nepal [18,19]. This gap limits our understanding of the compounded risks faced by women in these settings and the policy responses needed to address them. To our knowledge, most existing studies in Nepal focus on single determinants (e.g., transport, cost) or use SDH broadly, but intersectionality is almost absent in this context. We acknowledge that the only partial example is Khatri and colleagues’ work, which employs an intersectional lens for service coverage, rather than lived experiences [19]. We also note that the global reviews confirm that intersectionality is underused in perinatal health research [18]. Our study addresses this gap by applying the Social Determinants of Health framework and intersectionality to explore how geography, infrastructure, and poverty intersect to shape maternal health experiences in rural Nepal. Based on the evidence we are aware of, we also note that while limited research in Nepal has applied an intersectional approach to maternal health, this study contributes to this emerging area of inquiry.

## 2. Method

As the primary aim of the study was to explore the experience of pregnant women, we used a qualitative research design informed by a narrative approach, which is well-suited for exploring women’s lived experiences in depth. This approach enabled us to be more flexible and to use open-ended questions, allowing our participants to speak freely about what matters most in their pregnancy and their access to health services [20]. While the interview guide was developed, it was never imposed on participants to answer all the questions; instead, it was used to facilitate discussion of the interview’s main topics. A narrative approach was adopted in this research, which enabled participants to share their experiences in a story form, which is considered one of the most effective approaches to understanding people’s lived experiences.

### 2.1. Data Collection

The data were collected at Ilam District Hospital in the Eastern region of Koshi Province, Nepal. The hospital provides services to residents in rural, hilly, and remote areas. The women were approached in the maternity unit, where they were waiting to see doctors or receive test results. A combination of purposive and convenience sampling was used to recruit participants [21]. Purposive sampling ensured that the women selected were pregnant, aged 18 or above, and accessing maternal healthcare services at the district hospital, aligning with the aims of the study. Convenience sampling was applied to approach women who were available and willing to participate during their visit to the maternity unit. The inclusion criteria for the participants were:

- Pregnant woman at any stage of their pregnancy (regardless of the history of the number of births and pregnancies);
- Age 18 years and over;
- Living in the region and accessing maternal healthcare services at the District Hospital;
- Having the ability to give consent to participate in the study.

Before data collection, two one-to-one pilot interviews were conducted to avoid technical terminology related to maternal health and to ensure that the interview questions and other documents, such as information sheets and consent forms, were culturally appropriate. The average time for the in-depth one-to-one interviews was 30–45 min, but the researcher spent 10–15 min before and after each interview to build rapport, ensure participants were confident about the interview process, and allow them to reflect on the interview process. A total of 20 one-to-one interviews were conducted and data saturation was reached at the 17th interview, with the final three interviews confirming that no new themes emerged. This sample size aligns with qualitative research standards for achieving depth and diversity of experiences [20,22].

Due to the COVID-19 pandemic, and to ensure the safety of participants and researchers, interviews were conducted virtually, seated, between 25 September 2020 and 13 November 2020. Virtual interviews were one of the best ways at that time to collect data, considering the safety of participants and researchers [23]. All interviews were audio-recorded, although they were conducted on an audiovisual platform to ensure trust between the researcher and participants and to facilitate participants’ expression in case they were experiencing any distress during the interview. Both verbal and written consent were obtained before the interviews. The hospital supported arranging a phone that was kept securely, in accordance with the hospital’s policy. In the researcher’s virtual presence, the participants signed the consent form and reviewed the information sheet with the researcher’s assistance. Signed forms were scanned and submitted electronically via a secure platform. In accordance with hospital policy and ethical approval conditions, all physical copies of consent forms were destroyed to ensure confidentiality. This process ensured voluntary participation, protected participants’ privacy, and maintained the integrity of the research. The gatekeeper’s role was limited to connecting participants with the researcher and setting up the phone technology. All interviews were conducted in a designated room provided by the hospital, which had a lockable door, and no gatekeeper was present to maintain confidentiality and ensure a power balance.

### 2.2. Data Analysis and Interpretation

Data was analysed inductively using reflexive thematic analysis and also followed a six-step process, as discussed by Braun and Clarke [24,25,26], and NVivo 12 was used to organise the data. First, the researcher familiarised herself with the data by repeatedly reading transcripts and noting initial impressions. Second, initial codes were generated inductively to capture meaningful features related to maternal health experiences. Third, codes were organised into potential themes, followed by a systematic review of the supervisors to ensure coherence and relevance to the research questions. Fourth, themes were refined, defined, and named to reflect the essence of participants’ narratives alongside the supervisors of this research. Finally, illustrative tables were produced with example quotes for each theme to demonstrate transparency and rigour in interpretation. We also reflected on how the researcher’s insider positionality as a Nepali woman may have shaped rapport with participants and interpretation of the narratives. Reflexivity was maintained throughout the process, and NVivo 12 was used to manage coding and theme development. To ensure rigour and trustworthiness, several strategies were employed throughout the research process. The supervisors of this study closely monitored and verified the robustness of the process to ensure that the researcher’s biases had no influence and that the data accurately represented the participants’ voices. Peer debriefing sessions with the supervisory team were conducted to critically review emerging codes and themes, reducing the influence of researcher bias. Reflexivity was maintained through regular discussions, acknowledging the researcher’s insider perspective and its potential impact on interpretation. Reflexivity practice is also noted, as the first author of this research paper has experience in conducting qualitative research. The researcher and her supervisor were fully aware of their role, acknowledging their self-awareness and values on this topic, and efforts were made to engage critically and transparently with the emerging code using an inductive approach [26]. While the researcher’s insider status as a Nepalese woman brought valuable insights in this research, the supervisory team critically discussed the meaning and interpretation of emerging themes, which restricts the researcher from being influenced by personal assumptions [27]. Although coding was conducted inductively without imposing theoretical categories, the Social Determinants of Health (SDH) and intersectionality frameworks informed the interpretive stage of the analysis. These frameworks helped us understand how the inductively derived themes related to broader structural determinants and how multiple intersecting social positions shaped participants’ experiences.

### 2.3. Ethical Statement

Ethics Approval and Consent to Participate: Ethical approval for this study was obtained from the Nepal Health Research Council (Registration No: 356/2020) and Canterbury Christ Church University (Ref: ETH1920-0026). This research is guided by the ethical principles of autonomy, beneficence, non-maleficence, and justice during data collection and throughout data analysis and reporting [28]. The participants’ decisions were respected without any influence from the researchers’ perspective. All the participants were provided the opportunity to clarify any questions they may have before, during, and after the interviews. All participants provided informed consent prior to participation, including verbal and written consent, in accordance with ethical guidelines. To maintain confidentiality, we used pseudonyms, such as P1, to represent each participant. To ensure thematic flow, we used ellipses […] and removed irrelevant quotes.

## 3. Results

As the purpose of this paper was to present the experiences of rural women, only participants from rural areas were included. However, the author acknowledges that the broader PhD research involved women from both rural and urban settings. We also want to re-emphasise that the coding was conducted inductively and thematically; theoretical frameworks SDH and intersectionality were applied during the interpretive stage to contextualise and enrich the understanding of the emergent themes. In this research, Participants represented a range of sociodemographic backgrounds. The majority had more than one pregnancy, with parity ranging from first-time mothers to women experiencing their sixth pregnancy. Travel distance to the district hospital varied substantially: while some women lived within 30 min of the facility, most reported travelling more than one hour, often requiring additional walking time due to limited transport availability. These characteristics help contextualise the diverse experiences reflected in the findings.

**Figure 1:**
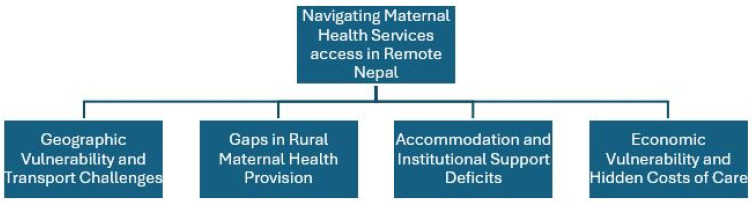
Navigating maternal health services in remote Nepal: Four major themes identified through thematic analysis.

Figure 1 presents the four major themes from the inductive thematic analysis, visually illustrating the compounded challenges faced by rural women. These are: Geographic Vulnerability and Transport Challenges; Gaps in Rural Maternal Health Provision; Accommodation and Institutional Support Deficits; and Economic Vulnerability and Hidden Costs of Care.

### 3.1. Theme 1: Geographic Vulnerability and Transport Challenges

Access to maternal healthcare in rural Nepal is often shaped by geography and the availability of transport infrastructure. For many pregnant women in rural Nepal, the journey to health facilities is long, uncomfortable, and unpredictable, particularly in the last stage of pregnancy. The women’s quotes in this section show how poor road conditions, unreliable transport services, and long travel times create significant barriers to timely, safe maternal care. Participant 7’s experience captures the compounded challenges of distance, weather, and unreliable transport systems. Her account reflects not just logistical difficulty but the emotional toll of navigating unsafe conditions while pregnant. According to her,

*I live far from this town 2–2:30 h by public van/taxi/jeep. Big Bus does not go to my village because the road is not in good conditions, it is a gravel road. It is raining too much, and flood on the road is everywhere. For the last 4–5 days, I was trying to come to this hospital but only today I got a vehicle so came for a video X-ray. We managed to come here today and hoping to return home by the same vehicle that returns to the village. We need to get in the vehicle at 4 pm to return home.*

*(P7)*

Her story is echoed by Participant 4, who describes how even reaching the nearest bus stop requires a long walk, and how seasonal changes further disrupt access. This highlights the fragility of rural transport systems and their impact on timely care. According to her,

*Commuting to this hospital is not easy for us. It takes walking 30 min to get the bus from my home, after getting the bus it takes about 2 h to reach this hospital. We do not have a public bus facility in the village. Even to reach the health post in my village I have to walk for half an hour. The public bus does not go to my village because the road is not good for the bus in my village. Today morning, we had to walk half an hour to get the bus to come here. Before, when the weather was good, and the road was better the bus service was available in our village.*

*(P4)*

Participant 11 adds a critical layer to this issue, describing how the lack of timely transport intensifies the physical discomfort of travelling on gravel roads during late pregnancy. Her reflections point to a broader systemic failure to prioritise maternal mobility in rural planning. According to her,

*I had to travel 2:30 h in a vehicle (public van) on the gravel road. I am heavily pregnant. A good road is important. I do not mind travelling long, but the gravel road is more problematic. I feel sometimes instead of gating in vehicles I would prefer walking but that is not possible. My home is far from this hospital. The roads have lots of bumps, potholes, cracks. That makes me tired and I feel very uncomfortable especially in this last stage of pregnancy. I think our government has not seen this yet. I hope our government can see it soon. […]. I am scared of traveling in vehicles that are more troubling, not comfortable at all, very bumpy. In addition, we do not get vehicles on time. We have to wait for the same vehicle that comes to this town in the morning and only that vehicle returns to my village. Sometimes it can be too late, it can be dark. […] I wish all pregnant women could reach the service on time. The road condition is bad. We do not have enough vehicles that provide services from our village to this town, and most of the time the transport service is not on time.*

*(P11)*

Together, these narratives revealed that transport-related barriers are not isolated incidents, but rather part of a broader pattern of infrastructural exclusion, calling for urgent policy attention to improve rural mobility, especially for pregnant women whose health outcomes depend on timely and safe access to care.

### 3.2. Theme 2: Gaps in Rural Maternal Health Provision

While geographical vulnerability contributed to gaps in service access, this theme focuses specifically on the limitations within rural health facilities rather than transport-related barriers. Beyond the transport system, the availability and quality of healthcare services in rural areas of Nepal remain significant concerns for pregnant women. Although local health posts offer basic care, the unavailability of diagnostic and treatment facilities means women have to travel to distant hospitals in the town or bigger health service providers. In this section, women shared their experiences with the limitations of rural health services and their desire for more comprehensive care closer to home. According to Participant 5, while transport remains a significant barrier, the limited scope of services available in rural health posts forces women to seek care in distant urban hospitals:

*I live far from this town (name of the village, hilly area). It takes 1 h by taxi to arrive at this hospital but half an hour walk to get the taxi. The public bus/taxi does not go near my home. […] I go for a health check-up every month to the health post near my home. They are ok. I like them. To this hospital, I came yesterday but I could not see the doctor. So I stayed in the town overnight at my relative’s place. Today I am here again. After completing all the check-ups I will go home today evening if possible. They did tell me everything about what to do. They said me to do a video-Xray next time. I need to come to this hospital for the blood test and video-Xray. We do not have all these services near my home, in the village health post.*

*(P5)*

Her experience is not unique. Participant 3 echoes similar concerns, emphasising how the lack of diagnostic services and poor road conditions delay access to care and increase risks during emergencies. Her reflections highlight the urgent need for investment in rural health infrastructure: According to her,

*Yes, we do have a health post near our home in the village. I used to go there for regular check-ups. But we do not have a video X-ray facility in the village. I wish all the services to have near to us. It is a very difficult road to commute to arrive at this hospital. Sometimes we can’t arrange vehicles on time. Many women’s lives would be saved if we had all the services in our village. We can’t come to this hospital immediately when we need health services because it is difficult to commute due to bad roads. If it is a rainy season, then it is more difficult. I wish health services could be available near us. I cannot come whenever I wish to come to this hospital. If it is an emergency, then it is difficult to manage to come to this hospital. I can’t come to this hospital when I need immediate health services due to bad roads and limited transport services.*

*(P3)*

Together, these accounts underscore the critical gap between policy and practice in rural maternal healthcare. While health posts exist, their limited capacity forces women into a cycle of delayed care and repeated travel, often at great personal cost. Addressing these gaps requires not only infrastructure upgrades but a rethinking of how maternal health services are distributed and accessed across Nepal’s rural regions.

### 3.3. Theme 3: Accommodation and Institutional Support Deficits

For many rural women, accessing maternal healthcare involves more than just reaching a hospital. It often requires temporary relocation to urban areas due to long travel distances, limited services at village health posts, and unpredictable availability of care in towns. These circumstances introduce additional emotional and financial burdens, especially for those without family support in urban settings. The lack of hospital-provided accommodation reflects a systemic gap in maternal health planning that fails to consider the lived realities of rural women. Participant 5’s account illustrates how the absence of accommodation services forces women to rely on relatives or incur extra costs, adding complexity to an already difficult healthcare journey:

*Sometimes we have to stay in the town for 2–3 days to complete all health check-ups. If I would not have relatives who live in the town then I had to stay in a hotel and that would cost more, for food and accommodation, etc. This hospital does not provide accommodation for us who come from far and rural hilly areas. I wish to have all the services near to my home that would be easily commutable. It’s difficult for me to come here to this hospital on my own. I need to have someone with me every time. That is also another problem to manage health check-ups timely.*

*(P5)*

Her experience is echoed by Participant 12, who describes the uncertainty of waiting for delivery while staying with relatives in town. Her reflections highlight the emotional strain of being away from home and the need for institutional support for rural women during late pregnancy:

*I am staying at my relative’s place. I came here 3 days ago and still waiting for my delivery. I don’t know how long I have to wait for delivery. My husband and father-in-law are at home. I came with my 5 years old son. As my relatives are here in this town, it is easier for me to come here for better health services rather than going anywhere else where I do not know anybody […] I wish all the services available to my local area. If these services were available, then I would prefer to live in my home until the last date of pregnancy and go for health services when needed. Now I came here to this town, staying with my relatives. I wish the government could provide accommodation in this hospital so that pregnant women can come earlier and stay few days until the delivery is completed. I do not know when the delivery will happen, how long I have to stay here. Traveling this long distance is a big problem during pregnancy. We do not have a reliable transport system.*

*(P12)*

Participant 3 further reinforces the need for accommodation support. Her temporary relocation due to health concerns shows how rural women often bear the cost of accessing care, both financially and socially:

*I live in a village, but for the last 15 days, I have been living in this town because I am having tummy pain. It takes 1 h by taxi; walking takes about 1.30 to 2 h. We live in a hilly area. Currently, I am living with my sister, and she has rented accommodation here in this town. The doctor called me today for a health check-up, so I am here today. We do not have a public bus service to come to this hospital from my home in the village.*

*(P3)*

These accounts collectively highlight a critical gap in maternal health policy, which is the absence of accommodation support for rural women. Without such provisions, access to care remains uneven and burdensome, reinforcing health inequities and placing additional stress on those already navigating complex social and economic challenges.

### 3.4. Theme 4: Economic Vulnerability and Hidden Costs of Care

Financial hardship is a recurring theme in the experiences of rural women seeking maternal healthcare in Nepal. The cost of transport, diagnostic tests, medication, and emergency referrals often exceeds what families can afford, especially for those relying on daily wages or subsistence farming. These financial pressures intersect with other systemic barriers, making access to care not only difficult but sometimes impossible. The following quotes illustrate how economic vulnerability shapes maternal health experiences and outcomes.

Participant 7’s account highlights how financial constraints, combined with transport challenges, place a heavy burden on rural women. Her reflections also point to the need for government-supported services to reduce out-of-pocket expenses:

*I wish government should give free ambulance services to pregnant women because transport is difficult for us who are living far from this town, especially in hilly village areas. Women like me have many problems. My husband works abroad, so I feel it is ok to complete health check-ups in terms of cost. But many women in my village cannot afford the cost of these tests. If the government would offer free services for all the blood tests and other health check-ups, then it would support me and other pregnant women who are in difficult financial situation. Women cannot buy even calcium tablets. If all the services and medicines are free then women will get better treatment in their pregnancies.*

*(P7)*

Building on this, Participant 11 expresses concern about the affordability of basic services and the cumulative cost of repeated visits. Her account underscores the need for financial protection mechanisms, especially for low-income families:

*I have concerns about how we are going to manage enough money we need for better treatment during the delivery. I wish even calcium tablets are given free of cost from the hospital. Video-Xray is a little costly. If it would be free of cost, then it would be a great relief for poor families like us. Last time, I paid about NRs 400 (£2.50 approximately) for video-Xray and blood tests. We are farmers. We need to arrange even that amount of money. Every time I have to spend money for travel and health check-ups such as blood tests and video-Xray in this hospital. It costs about Rs 2–3 thousand (£15–£25) every time I come to this hospital. Sometimes, travel is very expensive if we have to reserve a taxi to come. Many women may not have money for travel and health check-ups. It is difficult for me too. If we are recommended to have these basic health check-ups, we should get them free, especially for poor women during pregnancy. Sometimes, I think about how to manage everything in the future.*

*(P5)*

Participant 15’s reflections reveal how financial uncertainty intensifies the stress of potential complications, especially when emergency referrals are involved. Her account highlights the need for emergency financial support and better transport systems:

*In case my caesarean section has any complications, and I am referred to another hospital, it will be very expensive for us. The transport system is not good. In case of emergency, we need to arrange a private vehicle. It is costly, and I have that worry. […]. We do not know much about any other places. This is a government hospital, so I have the belief that it will not be very costly. But I cannot imagine going to another hospital from here. We are a poor family living in a village. My husband works on daily wages to feed the family. I cannot be prepared in advance for the additional expenses.*

*(P15)*

Like Participant 17, many women lack awareness about the scheme suggests that such initiatives may not be reaching rural populations effectively, limiting their potential impact:

*I do not know exactly what the benefit of this health insurance is’.*

*(P17)*

Participant 4 added another aspect in financial support, reliance on informal financial support and livestock income illustrates the adaptive strategies rural women employ to manage healthcare costs in the absence of formal support systems:

*My husband will arrange if we need to manage some money. I can also borrow from my sisters. My husband said he will arrange everything in case we need to go to a bigger hospital. so I do not worry about finance. […] I have feelings and worry that we are going to have a new baby and we need to manage our expenses and income. I think raising these cows and goats will help our finance.*

*(P4)*

These narratives collectively highlight the urgent need for inclusive financial protection policies, better outreach for existing schemes, and targeted support for rural families. Without these measures, economic hardship will remain a significant barrier to safe and equitable maternal healthcare.

## 4. Discussion

Based on the findings of this research, we understand that maternal health experiences in rural Nepal are shaped by a complex interplay of geographic, economic, and existing infrastructure and institutional factors. We aim to apply the understanding of the Social Determinants of Health (SDH) framework and the concept of Intersectionality to better understand how complex circumstances and structural barriers intersect with each other, presenting compounded risks to many pregnant women residing in rural areas of Nepal. These findings highlight Nepal’s challenges in meeting global commitments, such as the WHO’s Universal Health Coverage (UHC) and Sustainable Development Goal 3, which aim to achieve equitable access to maternal health services by 2030. Persistent gaps in health service provision, transport, accommodation, and financial protection indicate systemic weaknesses in translating policy into practice.

Access to transportation facilities is well recognised as one of the SDH that determines people’s health, which is visible in this research as poor road conditions, long travel distance, and unreliable public transportation significantly obstruct maternal health services of women in the region, which is well presented in this research and also acknowledged in the previous literature [29]. The infrastructure, such as transportation, reveals deeply rooted systematic inequalities in resource allocation by the government of Nepal, where people from rural areas are greatly disadvantaged in many aspects, including health services, due to limited and unreliable transport facilities. Looking at this issue through the lens of intersectionality, poor pregnant women from rural regions are likely to experience more vulnerability. The mental distress experienced by women highlights how socioeconomic and geographical isolation contribute to further marginalisation for women in the region. This could be one of the reasons maternal health services accessed by women from rural areas are lower in Nepal, as stated in the previous research [30]. The persistence of these barriers reflects structural issues, including inadequate decentralisation of services, weak local governance, and limited investment in rural infrastructure. These experiences reflect the interaction of multiple structural domains, including rural transport governance, health facility service organisation, and the absence of institutional support for accommodation, which together create specific gaps through which rural pregnant women fall.

Limited health service availability in rural parts of Nepal is evident in the previous research. However, this research has presented the amount of distress and complexity women experience during pregnancy due to limited service availability in the rural region. Social determinants of health explain that the availability of health services can shape health outcomes. Still, they are also greatly influenced by the social conditions in which people are born, live, and grow. The women in this research demonstrated a strong desire to access health services, often travelling miles away due to the limited availability of services at the nearby health post where they reside. From intersectionality perspectives, these systematic gaps can affect women differently based on their location, economic status and family support available to them. The women often travelling long distances and being delayed in care increased the health risk, and caused significant emotional and financial distress too, which is well discussed in the previous literature [31].

We understand that housing and institutional support are key determinants mentioned in the framework of social determinants of health. In this research, women’s experience of a lack of institutional support for accommodation during pregnancy when they come for an antenatal health check-up causes significant distress to many women, as they may not have anyone to offer accommodation in the town. The absence of maternity waiting homes (MWHs) means that women have to rely on their relatives’ goodwill or spend additional money on accommodation and food, in addition to accessing health services. From an intersectional perspective, it can be argued that some women are disproportionately affected by geographical isolation and lack of social support, and economic hardship [7]. The concept of MWHs is not new, and there is evidence of the success of MWHs in increasing access to maternal healthcare in Nepal [32]. Despite this evidence, Nepal is still slowing the process to provide MHWs to improve the maternal healthcare access to all women, regardless of geographical and socioeconomic background. The absence of maternity waiting homes, despite their inclusion in Nepal’s Safe Motherhood Roadmap [12], illustrates a disconnect between policy recognition and operationalisation.

In this research, financial hardship and costs associated with maternal health service access were highly spoken of by the women. Financial circumstances are one of the key social determinants of health, as discussed in the literature, highlighting that income within socioeconomic status is a significant factor in health equity [33]. Despite some financial support for maternal health services within Nepal’s health system, women in remote areas still face significant barriers to accessing these services, which raises questions about the implementation of the Safe Motherhood Programme in Nepal [34]. Women are finding that financial hardship is one of the significant factors contributing to insecurity and vulnerability among them. From an intersectional perspective, financial hardship further intersects with gender, geography and awareness of the insurance system, putting the women at further risk. The health insurance scheme could offer some support, but it is not free of cost. As a result, many women may struggle to afford it, and most importantly, women from rural regions may not be aware of it [35,36]. The finding suggests that financial vulnerability intersects with gender, geography, and awareness gaps, creating compounded disadvantages for rural women. This intersectional lens reveals that policy interventions focusing solely on cost reduction without addressing social determinants will remain insufficient.

Overall, our findings highlight that maternal health challenges in rural Nepal are not isolated but rooted in broader systemic inequities. While Nepal has adopted policies such as the Safe Motherhood Programme and recognised maternity waiting homes in its strategic roadmap, implementation remains uneven. This gap contrasts sharply with global frameworks like WHO’s Universal Health Coverage and SDG 3, which advocate for equitable, timely, and quality maternal care. Addressing these systemic factors requires integrated strategies that go beyond clinical interventions and prioritise tackling social determinants and intersectional vulnerabilities.

## 5. Limitations of the Study

We acknowledge that this study has some limitations. First, the sample size was relatively small (20 participants), which, while sufficient for achieving data saturation and capturing diverse experiences, limits the breadth of perspectives. Second, interviews were conducted virtually due to COVID-19 restrictions, which may have affected rapport, and the depth of non-verbal communication compared to face-to-face interviews. To overcome this, the researcher invested additional time in building trust before and after interviews and used video calls to maintain visual cues wherever possible. Third, as a qualitative study focused on one district hospital in eastern Nepal, the findings may not be fully transferable to other regions with different cultural, geographic, or health system contexts. We also recognise that the use of purposive and convenience sampling, combined with recruiting participants exclusively from a district hospital, may introduce selection bias by excluding women who face the most severe barriers and are therefore unable to reach the facility. However, thick descriptions of context and detailed participant narratives were provided to enhance transferability. We included the voices of women from rural regions only, as the primary purpose was to understand their experiences. This paper focuses exclusively on the experiences of rural women, whereas the author’s PhD research included participants from both rural and urban areas. This narrower scope was chosen to align with the specific aim of this study. Therefore, we do not claim that these findings represent the experiences of women across the entire country of Nepal, and the findings should be interpreted within this specific context.

## 6. Conclusions

These lived experiences of women in this research illustrate how maternal health in rural Nepal is shaped not only by medical factors but by a broader context of infrastructural neglect, economic vulnerability, and policy gaps. By applying the Social Determinants of Health (SDH) framework and the concept of intersectionality, this research allowed us to understand how multiple layers of disadvantage, such as geographical isolation, lack of an adequate transportation system, financial hardship, and limited institutional support, intersect and present a compounded risk for pregnant women in the region. The findings also highlight that these challenges experienced by the pregnant women are broader systemic structural inequality that exists in Nepal. This research concludes that these barriers not only restrict access to essential maternal health services but also contribute to emotional distress and marginalisation, particularly for women with limited social and economic resources. Addressing these challenges requires an integrated approach, such as investment in rural transport and health infrastructure, expansion of diagnostic and emergency services at the local level, provision of accommodation support for expectant mothers, and the implementation of inclusive financial protection policies. We conclude that by centring the voices of rural women and responding to their articulated needs, Nepal can move toward a more equitable and effective maternal healthcare system. Therefore, we argue that this study offers an important and original contribution to the literature by clearly illustrating how deep-rooted structural determinants, together with intersecting social positions, intersect to shape and intensify rural women’s lived maternal health experiences.

## 7. Recommendations

Based on the findings and discussion of this research, we recommend the following four steps to guide future practice and policy, while recognising that these recommendations are grounded in the lived experiences of participants and therefore represent strong, context-specific interpretative insights. These are:

1. Strengthen Transport Infrastructure and Awareness of Incentives: Based on the women’s experiences of transport and geographical context, we recommend an improved transportation system recognised within the policy of maternal health, as well as ensuring awareness of the existing incentives for transport expenses within the Safe Motherhood Programme among women to ensure timely and affordable transport for pregnant women.
2. Expand and Equip Nearby Health Facilities: We recognise and recommend that it is essential to expand health services to the nearby hospital. It includes ensuring the consistent availability of skilled birth attendants and crucial maternal health services that decrease the inequality gap currently existing in the health system of Nepal.
3. Implementation of Maternity Waiting Homes (MWHs): Based on the narratives of women, we highly recommend the implementation of Institutionalised Maternity Waiting Homes (MWHs) within Nepal’s national maternal health framework, ensuring sustainable funding and clear operational guidelines.
4. Enhance Financial Support and Insurance Accessibility: We emphasise the importance of financial support and insurance awareness. We highly recommend strengthening awareness campaigns about the social health insurance scheme, especially in rural and marginalised communities, and argue for subsidising or offering free enrolment for low-income families to reduce financial barriers to maternal care.

## Data Availability

All relevant data supporting the findings of this study are included within the manuscript.
